# The Validation of a Hemagglutination Inhibition Assay That Detects Antibodies Against a Newcastle Disease Virus-Based Vaccine Vector in Human Serum Samples

**DOI:** 10.3390/vaccines13040342

**Published:** 2025-03-22

**Authors:** Milton Nieto-Ponce, Edgar Reyna-Rosas, Rosa Andrea Palencia-Reyes, Carlos Blancas-Ruíz, Guadalupe Aguilar-Rafael, Marlenne Paola Rubicer Rubio-Diaz, Luis Alfonso Ramírez-Martínez, Claudia Carranza, Bernardo Lozano-Dubernard, Martha Torres, Horacio Zamudio-Meza

**Affiliations:** 1 Laboratorio de Inmunobiología de la Tuberculosis, Instituto Nacional de Enfermedades Respiratorias “Ismael Cosío Villegas”, Mexico City CP 14080, Mexico; miltoonnietoo.66@gmail.com (M.N.-P.); ereyna5@gmail.com (E.R.-R.); andypal93@gmail.com (R.A.P.-R.); carlosblancasruiz@gmail.com (C.B.-R.); carranza.salazar.claudia@gmail.com (C.C.); 2 Diagnósticos Clínicos Veterinarios, S.A. de C.V. (DCV), Mexico City CP 09810, Mexico; guadalupe.aguilar@dcvlab.com (G.A.-R.); marlene.rubio@dcvlab.com (M.P.R.R.-D.); 3 Laboratorio Avi-Mex, S.A. de C.V. (Avimex^®^), Mexico City CP 09810, Mexico; luis.ramirez@avimex.com.mx (L.A.R.-M.); lozano@avimex.com.mx (B.L.-D.)

**Keywords:** NDV-vectored vaccine, HI assay, validation of analytical methods

## Abstract

Background: An NDV-based vector has been used as a veterinary vaccine and, recently, as a human COVID-19 vaccine. However, data for the potential immune response against the vector in humans are scarce; therefore, it is important to evaluate the levels of antibodies produced. The HI assay is the gold standard for assessing the humoral response against NDV in poultry serum. Objective: Here, the objective was to validate the HI assay against the NDV-vectored vaccine to analyze antibodies in human serum. Methods: First, we standardized the conditions in human sera before validation. Results: The results for analytical performance in terms of selectivity, sensitivity, specificity, and positive and negative predictive values, as well as positive and negative diagnostic reliability, indicate that the assay is highly selective, allowing clear discrimination between positive and negative samples. Regarding repeatability and intermediate precision, we demonstrated that the assay has the precision to obtain consistent results, guaranteeing their reliability and truthfulness. Finally, the results regarding accuracy, linearity, and robustness indicate that the assay is accurate across the evaluated concentration intervals, with a linear correlation between low and high levels, and demonstrate that it is robust and consistent when serum–antigen interaction times are changed. Conclusions: We conclude that the suitability of the analytical method for its intended use is confirmed, guaranteeing the reliability of the results obtained under the established operating conditions.

## 1. Introduction

The Newcastle disease virus (NDV) is a pleomorphic virus measuring between 150 and 350 nm in diameter, and it can be found as spherical or filamentous particles. It is an enveloped, non-segmented virus from the Paramyxoviridae family and the subfamily Avulavirinae [1]. Its genome is a single-stranded, negative-sense RNA molecule which measures between 13 and 19 Kb and encodes six structural proteins—nucleocapsid protein (NP), phosphoprotein (P), matrix protein (M), fusion protein (F), hemagglutinin–neuraminidase (HN), and polymerase (L)—along with two non-structural proteins (V and W). This virus is the etiological agent of Newcastle disease, a highly contagious disease which mainly affects birds. It can manifest in three clinical forms in chickens: mild, caused by lentogenic strains; moderate, caused by mesogenic strains; and severe, caused by velogenic strains, representing an economic problem in the poultry sector [2,3].

NDV is categorized as a hemagglutinating virus due to the interaction of the HN protein on its surface with sialic acid residues present in the glycoproteins of red blood cells (RBCs) from chickens, pigs, guinea pigs, and humans, among others. This interaction leads to the formation of a network, called hemagglutination, which can be evaluated in vitro and is dependent on the affinity of the viral proteins for the receptors on the surface of RBCs, as well as on temperature, interaction time, concentration, and the origin of erythrocytes [4,5,6].

Lentogenic strains such as LaSota and B1 have low virulence and propensity for recombination, in addition to eliciting a strong immune response, making them suitable as biotechnological tools, for example, as an oncolytic therapy due to their immunomodulatory properties and high replication in tumor cells. They are also used as virus-based vaccine vectors or as vaccine strains in the poultry sector [7,8,9,10]. In the case of poultry, it is necessary to ensure the efficacy of vaccination by evaluating antibodies against NDV in vitro, as this prevents the spread of the virus.

The gold standard for the detection of antibodies against hemagglutinating viruses is the hemagglutination inhibition (HI) technique, which involves serial dilutions of a serum sample and contact with the NDV antigen, preventing the binding of the HN protein in the presence of RBCs, causing them to sediment and form a button with well-defined edges at the bottom of the well that slides in a drop shape when the plate is tilted at 45°. The concentration of antibodies is defined by the maximum dilution at which the button movement is observed [4].

There is evidence of the use of the HI assay to evaluate the immune response against NDV in humans, given that this virus can potentially cause zoonosis, the symptoms that may occur are tearing, irritation, and swelling, although these are generally self-limiting and are not considered a health risk [11]. Therefore, this technique can be used to evaluate the immune response to an NDV-vectored vaccine, which is of great importance, as it has been reported that the use of Adenovirus (Ad)-based vaccines, such as Ad5 in repeated immunizations, affects the organism’s effectiveness in producing antibodies against the antigen of interest due to high antibody production against the vector, which may condition its usefulness [8,9,12,13]. On the other hand, the use of NDV as a vaccine vector is safe, and a low response to the vector is induced by producing low antibody titers, as seen in the case of the AVX/COVID-12 “Patria” vaccine approved by the Comisión Federal para la Protección contra Riesgos Sanitarios (COFEPRIS) in Mexico [12].

To use this technique for the analysis of human sera, it is necessary to standardize the conditions of each parameter involved, such as the minimum amount of antigen capable of causing hemagglutination, expressed in hemagglutinating units (HU) [14,15]. Additionally, the nature of serum samples is an important factor to consider, as mammalian serum samples, unlike avian serum, contain nonspecific inhibitors that can adhere to the surface of RBCs, potentially causing false positive results [15,16,17]. Some research groups have proposed strategies to reduce the presence of nonspecific inhibitors of hemagglutination: the heating of sera to high temperatures (56–65 °C), pretreatments with a kaolin solution, the pre-absorption of serum samples in an RBC suspension, or the use of receptor-destroying enzymes (RDEs) such as filtrates derived from *Vibrio cholerae* [16,18,19]. Although the HI assay is an established test that has not undergone substantial changes, there are still no reports in the context of the human NDV vaccine vector within an analytical validation process aligned with international regulations.

In this work, we present the standardization and validation of an HI assay that allowed the evaluation of the immune response against the NDV vector in human sera from volunteers vaccinated against SARS-CoV-2 with the AVX/COVID-12 “Patria” vaccine, demonstrating the suitability of the analytical method for its intended use. We also confirm that it guarantees the reliability of the results obtained under the established operating conditions.

## 2. Materials and Methods

### 2.1. Reagents

The reagents used were RDE *Vibrio cholerae* Filtrate Solution (Sigma-Aldrich C8772-IVC; Burlington, MA, USA) and 25% kaolin solution in 1× PBS, pH 7.4 (Gibco REF 10010-031, Waltham, MA, USA). The conditions of use of each reagent are described in subsequent sections.

### 2.2. Newcastle Disease Virus (NDV) Antigen

**Figure 1:**
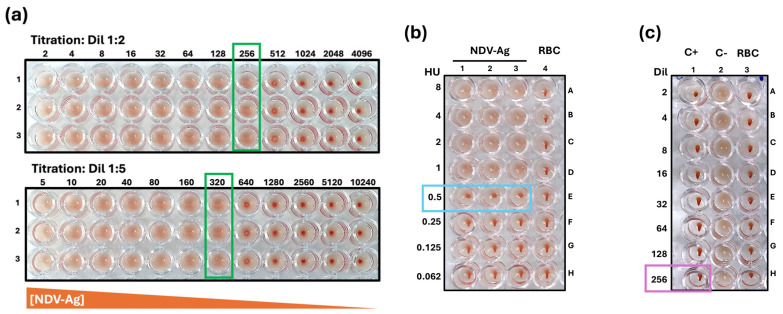
Representative images of NDV antigen titration, control of HU for HI assay, and controls of HI assay. (**a**) NDV antigen was diluted 1:2 and 1:5 in triplicate (rows 1, 2, and 3), and the titration was assessed by serial dilutions. The titer is the last dilution where hemagglutination of the RBCs is observed (green box). The orange triangle under the box represents the concentration of NDV antigen. (**b**) The control test HU for IH assay. The NDV antigen suspension at 8 HU is loaded in positions A1 to A3, and serial two-fold dilutions were performed (8, 4, 2, 1, and 0.5 HU); 0.5 HU indicates that the agglutination is partial (light blue box). In the column from A4 to H4 is the RBC integrity control. (**c**) Controls used in HI assay: positive control (C+)—hyperimmune serum from pathogen-free chicken immunized with the attenuated NDV vaccine (A1–H1), with an established titer of 1:256 (purple box); negative control (C−)—non-immunized serum from pathogen-free chicken (A2–H2); integrity control of the RBC suspension from pathogen-free chicken obtained from peripheral blood, used as a read-out system (A3–H3). The integrity of the RBCs was determined with the test of the displacement of the precipitate, forming a “teardrop” when the plate was tilted at an angle of 45°, used as part of the HI trial acceptance criteria.

The NDV antigen LaSota strain was propagated in pathogen-free chicken embryos and inactivated with 3.7% formaldehyde solution (Batch 23920102, Diagnósticos Clínicos Veterinarios, S.A. de C.V., Mexico City, Mexico). The concentration is expressed in hemagglutinating units (HU, titer of 1:256). This batch was used for all tests performed. Before the start of each series of tests, the HU titer of the NDV antigen was confirmed by titration, with the test also being performed for the HU control, following the instructions of the protocol established by the supplier (Figure 1a,b).

### 2.3. Red Blood Cell (RBC) Suspension

A 5% suspension of chicken RBCs prepared in 1X PBS *v*/*v* with a certificate of analysis issued by the DCV company was used (Batch 23-08, Mexico City, Mexico). Pig RBCs were donated by the “Instituto Nacional de Enfermedades Respiratorias, Ismael Cosío Villegas (INER)” Biotherium (through its research protocol code B18-20), and human RBCs were obtained from volunteers with O-blood type (protocol code: C49-22); in both cases, a 5% suspension in 1X PBS *v*/*v* was prepared from whole blood following the guidelines of good laboratory and biological safety practices.

### 2.4. Serum Samples

Given the limitation in obtaining a sufficient number of NDV-positive human serum samples, we followed an experimental strategy for the generation of samples with a human serum matrix enriched with NDV-positive and -negative pig sera included in pre-clinical protocol.

Assay standardization: One serum sample from a human volunteer vaccinated against SARS-CoV-2 (AVX/COVID-12), obtained 21 days post-vaccination, was used as an exposure control for NDV (protocol code: C49-22). One serum sample from a pig vaccinated against SARS-CoV-2 (AVX/COVID-12) was used as a positive control for NDV, with a known HI titer of 1:512 (protocol code: C24_42), and human serum (Batch 21C0457, Valley Biomedical, Winchester, VA, USA) was used as a negative control for NDV.

Assay validation: Commercial pathogen-free human serum (Batch 21C0457, Valley Biomedical, VA, USA) was used as a human serum matrix during the experiments; 10 serum samples from volunteers who had recovered from influenza and 10 serum samples from volunteers who had recovered from COVID-19, one and two months after diagnosis confirmation, were used for the selectivity test (pre-pandemic control and unrelated viral respiratory disease). Four serum samples from pigs were also included: three positive samples against SARS-CoV-2 (vaccinated with AVX/COVID-12), with known HI titers of 1:64, 1:32, and 1:256, and one sample without vaccination as a negative control (protocol code: C24_42).

### 2.5. Test Controls

Positive and negative controls used during the tests were produced by the DCV company (with a certificate of analysis). A chicken serum immunized with the NDV vaccine (LaSota) from Batch 2301, which has a known HI titer of 1:256, and a chicken serum without an HI titer from Batch 2301 were produced using pathogen-free farm chickens (Figure 1c).

### 2.6. Standardization of Experimental Conditions for HI

**Figure 2:**
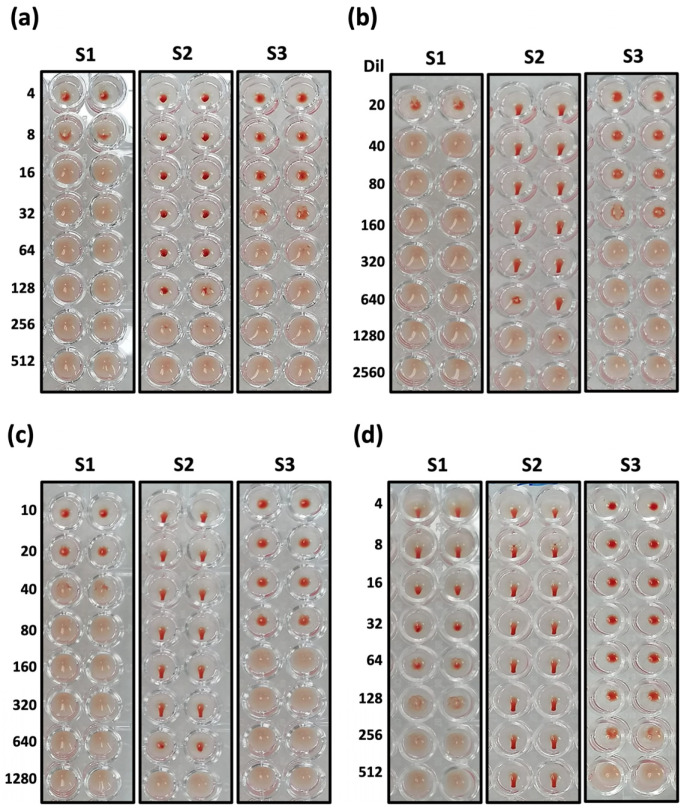
The effects of pretreatments on serum samples in the HI assay: (**a**) 1X PBS in 1:1 *v*/*v* ratio at 4 °C for 16–18 h (initial dilution of 1:4); (**b**) 5% RDEs and 5% RBCs in 1:5:4 *v*/*v*/*v* ratio at 4 °C for 16–18 h (initial dilution of 1:20); (**c**) 25% kaolin solution and 5% RBC in 1:2:2 *v*/*v*/*v* ratio at 4 °C for 16–18 h (initial dilution of 1:10); (**d**) absorption with 5% RBCs in 1:1 *v*/*v* ratio at 4 °C for 16–18 h (initial dilution of 1:4). (S1) Human serum sample vaccinated against SARS-CoV-2 with AVX/COVID-12, (S2) pig serum sample vaccinated against SARS-CoV-2 with AVX/COVID-12, and (S3) human serum sample negative for NDV.

Three concentrations of RBC suspension (1, 1.3, and 2%) from chickens, pigs, and humans were prepared to evaluate the suitable conditions for plate reading (sedimentation time and temperature). The plate reading is obtained when the RBCs form a circular button with defined edges at the bottom of the plate and slide in the form of a “teardrop” or “droplet” when the plate is tilted at 45°. The pig and human serum samples were inactivated at 56 ± 1 °C for 30 ± 5 min in a water bath and then subjected to four treatments: (a) PBS 1X 1:1 *v*/*v* was added and incubated at 4 °C for 16–18 h; (b) RDEs and 5% chicken RBC suspension in a 1:5:4 *v*/*v*/*v* ratio were added and incubated at 4 °C for 16–18 h, and in the end, samples were inactivated again; (c) 25% kaolin suspension and 5% chicken RBC suspension in a 1:2:2 *v*/*v*/*v* ratio were added and incubated at 4 °C for 16–18 h; (d) 5% chicken RBC suspension at a 1:1 *v*/*v* ratio was added and incubated at 4 °C for 16–18 h (Figure 2).

### 2.7. Experimental Strategy for the Validation of the HI Assay

We used a modification of the assay developed by the Food and Agriculture Organization of the United Nations (FAO) [19], which was obtained by our laboratory through a technology transfer agreement with the DCV company. This assay is considered a serological assay that is semi-quantitative, not normalized, and a non-pharmacopeial method. The protocol is briefly described below, based on the use of serum samples from farm chickens vaccinated against NDV. In 96-well “U” bottom plates, add 1X PBS; place the problem samples and test controls in the assigned column of the plate following a sequential order. Mix and perform serial dilutions by transferring an equal volume to the next column. Repeat this step until the last column is reached. Add the NDV antigen, previously adjusted to 8 HU, to all the wells of the plate and incubate at room temperature (19–25 °C) for 30 min (for antigen–antibody interaction). Finally, add the 1% chicken RBC suspension, incubate for 40 min at room temperature, and proceed to plate reading. The results are expressed as the highest serum dilution that presents an inhibition of hemagglutination.

### 2.8. Parameters and Validation Criteria

To support the validation process of the hemagglutination inhibition assay, we aligned our procedures with national and international guidelines for the adequacy and validation of analytical methods (Eurachem, Eurolab, 2016; ICH-Q2 (R2) Guidelines, 2018) [4,6,20,21,22,23]. To evaluate the analytical performance of the technique, we calculated qualitative parameters—selectivity, sensitivity, specificity, positive predictive value (PPV), negative predictive value (NPV), positive diagnostic reliability (PDR), and negative diagnostic reliability (NDR)—using contingency tables. These parameters represent analytical behavior, concerning the rate of true positive and negative data, as well as false positive and negative data, and are essential to understanding the errors associated with the method (systematic error) and their effect on the results. The established acceptance criteria were as follows: selectivity (χ^2^ calculated greater than χ^2^ from tables, degrees of freedom = 1, and α = 0.05); sensitivity (a/(a + c) ≥ 0.95); specificity (d/(d + b) ≥ 0.95); PPV (a/(a + b) ≥ 0.95); NPV (d/(d + c) ≥ 0.95); PDR (a/(a + b) ≥ 0.95); and NDR (c/(c + d) < 0.05). Repeatability and intermediate precision (coefficient of variation (CV) ≤ 20% and t-test with a *p*-value < 0.05) were also calculated, as well as accuracy (recovery percentage = 80–120% and CV ≤ 20%), linearity (linear regression with R^2^ ≥ 0.90), and robustness (different interaction time (serum–antigen): recovery percentage = 80–120% and CV ≤ 20%).

### 2.9. Analysts

Based on the validation design, two analysts were considered for the development of the experimental procedures. Both were trained and demonstrated that they had the skills required to develop the test in line with good laboratory practices and international standards such as ISO 9001:2015 [24] and ISO/IEC 17025:2017 [25].

To evaluate the qualitative parameters of the assay, 20 samples of an unrelated viral respiratory disease enriched with pig serum from both negative and positive samples for NDV at different levels of HI titers were used. Repeatability, intermediate precision, and accuracy were evaluated by two analysts on different days analyzing the same three samples. The samples analyzed were commercial human serum enriched with pig serum positive for NDV at three different levels of HI titers: 1:4 (low), 1:32 (middle), and 1:128 (high). The linearity parameter was evaluated by one analyst with three replicates of six human serum samples enriched with positive pig serum, obtaining HI titers calculated at three levels of quantification: 1:4 and 1:8 (low), 1:32 and 1:64 (middle), and 1:128 and 1:256 (high). Robustness was evaluated by one analyst with 30 replicates of a sample of human serum enriched with positive pig serum, obtaining HI titer values calculated at 1:128, with the samples subjected to three different antigen–sample incubation times (15, 30, and 45 min). Each result was obtained from samples evaluated in triplicate.

### 2.10. Statistical Treatment

The results obtained from each assay were expressed in HI titer and logarithm base 2 (Log2). The statistical treatment consisted of obtaining the average, standard deviation (SD), and coefficient of variation (CV%). The *t*-test and linear regression analysis were conducted with the help of the statistical program GraphPad Prism Ver. 10.1, according to the requirements of each aspect of the validation.

## 3. Results

### 3.1. Standardization of Test Conditions

The HI serological test is mainly used to evaluate antibody-mediated immunity against NDV or influenza in birds. In human samples, its use has only been reported for influenza [6,8,9], and only one report for NDV could be found [11]. This is why we standardized and adapted several parameters of the original technique. The origin of the RBCs is a critical factor that can influence the results, so we evaluated different types of RBCs (chicken, pig, and human), RBC concentration (percentage %), ambient temperature (two temperature intervals), and sedimentation times during the standardization of the assay. The results obtained showed that the sedimentation time of the pig RBCs was significantly more than 1 h, regardless of the concentration percentage and ambient temperature. In contrast, the chicken and human RBCs showed sedimentation times lower than 1 h at percentages between 1 and 1.5%. However, at 17–19 °C, the time for sliding at the bottom (teardrop) was higher than at 20–25 °C. Therefore, the 20–25 °C range was established for the validation of the test, and the percentages of the RBC suspension were between 1.3 and 1.5%, since the button was better defined and moved properly at 20–35 s (Table 1).

Another important element to consider is the presence of nonspecific inhibitors of hemagglutination in mammalian serum samples; therefore, we evaluated the effect of pretreatment in eliminating them [14,18]. When HI was performed using samples without pretreatment (human and pig serum), the samples did not agglutinate, forming a homogeneous network; instead, partial precipitation without displacement was observed. This phenomenon is known as “spongy buttons or partial agglutination”. However, once the serum was diluted, the spongy button became more discreet until it disappeared in positive and negative human samples and formed the expected hemagglutination network. In contrast, in positive pig serum samples, complete sedimentation was observed but not teardrop formation (Figure 2a). When comparing pretreatments between kaolin and RDEs with PBS 1X, no differences were observed in terms of the formation of spongy buttons in human samples (Figure 2b,c), unlike what was found in the pig samples, where teardrop formation was observed (HI titer 1:320). Meanwhile, with the pretreatment using 5% chicken RBC suspension, we found teardrop formation in both positive samples (HI titers 1:8 and 1:512), and partial agglutination was still observed (Figure 2d). Therefore, we conclude that the most appropriate pretreatment was the adsorption with 5% chicken RBCs for 16–18 h and 2–8 °C, starting with an initial concentrated dilution of 1:2 and a final plate dilution of 1:4 (Figure 2d(S1)).

### 3.2. Validation

**Figure 3:**
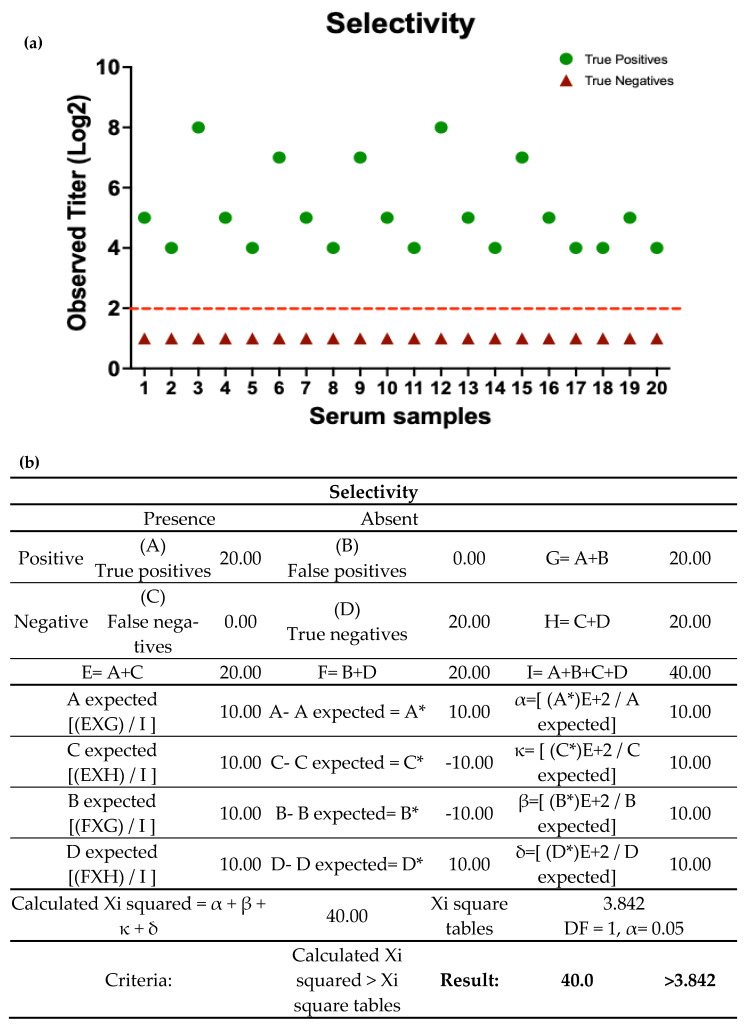
Analytical performance of HI assay. The results for the experimental evaluation of the qualitative selectivity parameter were obtained by one analyst from 20 samples of an unrelated viral respiratory disease enriched with pig serum negative for NDV and positive for NDV at different levels of HI titers. Each result was obtained from samples evaluated in triplicate. (**a**) Representative graph of the titers obtained in Log2 of the samples analyzed, indicating the cut-off point of 2 established with the positive samples. (**b**) Summary of the calculations for the evaluation of analytical selectivity using the Xi2 statistical test. * Indicates the result of subtracting the observed values from the expected values in each case.

To demonstrate that the assay is capable of detecting or measuring a particular analyte or particular inhibitory biological activity, parameters of analytical performance such as selectivity, sensitivity, specificity, PPV, NPV, PDR, and NDR were evaluated. The results obtained by one analyst from 20 samples of an unrelated viral respiratory disease enriched with pig serum (negative for NDV and positive for NDV at different levels of HI titers) showed inhibitory activity of hemagglutination in 100% of samples enriched with positive pig serum with HI titers between 4 and 8 (Log2), while the negative samples did not exhibit any inhibitory activity. These results indicate that the assay is highly selective under the experimental conditions used, as it allows for clear discrimination between positive and negative samples (Figure 3, Table 2 and Table 3).

The results obtained for the parameters of sensitivity, specificity, PPV, NPV, PDR, and NDR showed that the assay meets the established performance criteria. The absence of false positives and negatives, as well as values greater than the established criterion of 0.95, confirms the consistency of the assay (Table 2 and Table 3).

**Figure 4:**
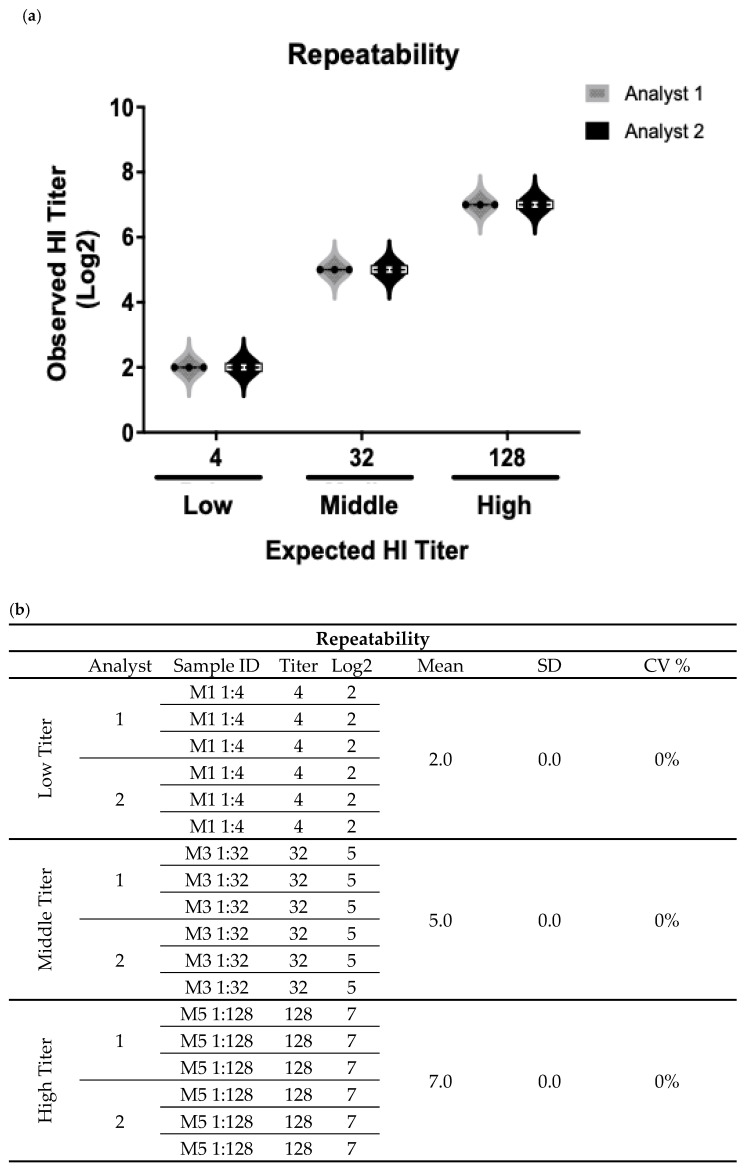
Results for repeatability of HI assay. The results were obtained by two analysts under the same analysis conditions on the same day from three human serum samples enriched with pig serum positive for NDV at three different levels of HI titers: 1:4 (low), 1:32 (middle), and 1:128 (high). Each result was obtained from samples evaluated in triplicate. (**a**) Representative graph of the average values obtained by each of the analysts for the different concentrations analyzed. (**b**) Summary table of the expected and obtained results at the three levels of titers for each analyst, as well as the values obtained for the average, SD, and CV.

Based on the guidelines for the validation of analytical methods using statistical parameters, repeatability represents the distribution of results from repeated independent experiments of a sample under specific conditions. In this regard, the results obtained by the analysts, analyzed independently on the same day, showed a standard deviation (SD) and coefficient of variation (CV%) equal to 0 at the three levels of quantification evaluated. The Student’s *t*-test showed that there are no statistically significant differences between the results obtained by each analyst, indicating that the test has the necessary precision to obtain consistent results from the same sample with an absence of inter-analyst variability (Figure 4).

**Figure 5:**
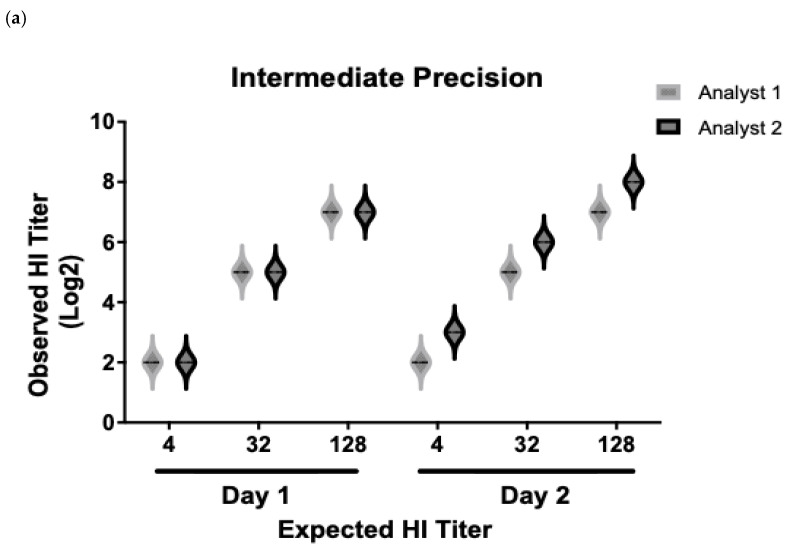

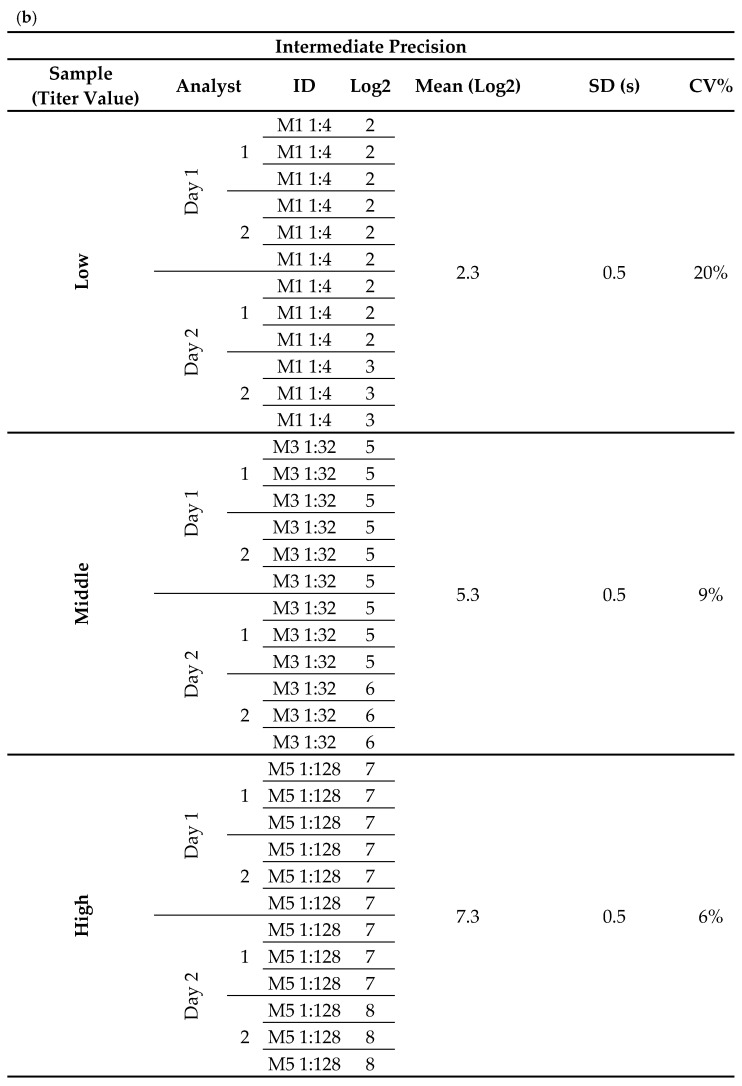
Results for intermediate precision of HI assay. The results were obtained by two analysts under the same conditions on different days from three human serum samples enriched with pig serum positive for NDV at three different levels of HI titers: 1:4 (low), 1:32 (middle), and 1:128 (high). Each result was obtained from samples evaluated in triplicate. (**a**) Representative graph of the average values obtained by each of the analysts for the different concentrations. (**b**) Summary table of the expected and obtained results at the three levels of titers for each analyst, as well as the values obtained for the average, SD, and CV%.

The next parameter to evaluate was intermediate precision; this represents the relative agreement obtained when evaluating independent results under variable conditions in the same laboratory. The results obtained by Analyst 1 maintained a CV% of 0 at the three levels of quantification. Regarding the analysis of the data obtained by Analyst 2, an increase of one logarithm was observed at the lower titer level on the second day of analysis. Despite the fact that the CV% observed was higher at the three evaluated levels (low: 20%; medium: 9%; high: 6%), these values remained below the established acceptance criteria of the test. This analysis was complemented by the application of the *t*-test, which demonstrated that there are no significant differences between the results of both analysts, obtaining a *p*-value equal to 0.712. This confirms that the behavior of the results obtained under the established experimental conditions is sufficiently consistent, which guarantees their reliability and truthfulness (Figure 5).

To assess the agreement between the observed values and the expected values using the method, the accuracy of the assay was calculated by determining the recovery percentages at three concentration levels. The results obtained showed individual recovery percentages of 113, 105, and 104%, and CV% values of 20, 9, and 6% for the low, medium, and high levels, respectively. In none of these cases were the established acceptance criteria for the test exceeded. These results indicate that the assay maintains its accuracy and precision in the different concentration intervals evaluated.

**Figure 6:**
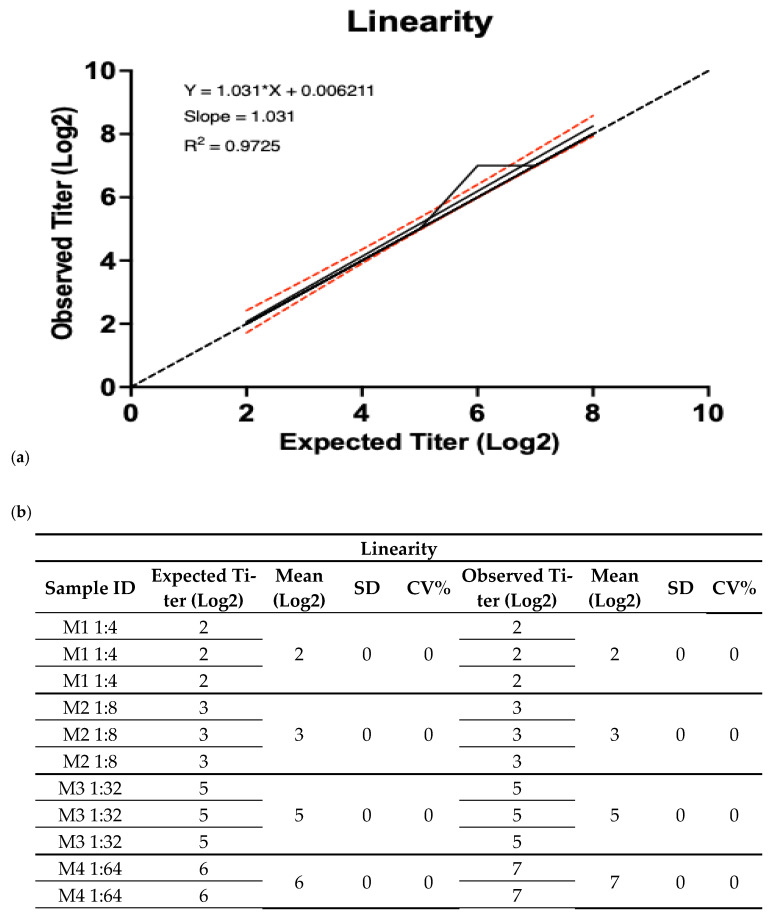

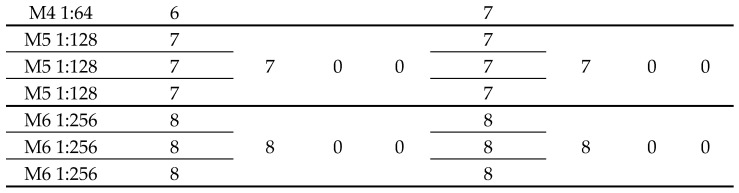
Results for linearity parameter of HI. The results were obtained by one analyst from six human serum samples enriched with positive pig serum, obtaining HI titer values calculated at three levels of quantification: 1:4 and 1:8 (low), 1:32 and 1:64 (middle), and 1:128 and 1:256 (high). Each result was obtained from samples evaluated in triplicate. (**a**) Representative graph of the linear regression test between the expected and observed values of the HI titers, presenting the coefficient of determination (R^2^) and the graph formula. (**b**) Table of expected and obtained results at the different titer levels from the assay and the obtained statistical values.

To ensure that the results obtained were directly or by mathematical transformation proportional to the concentration or inhibition titer present in the positive sample, the linearity of the assay was determined. The results obtained corresponded to those expected when observing a linear correlation between the low and high levels; however, at the middle level, a logarithmic increase in the observed values compared to the expected HI titer of 1:64 was observed. Despite this, the general linear trend was maintained with a coefficient of determination (R^2^) of 0.9725 and a CV% of 0 at each of the points. These results demonstrate that the established assay complies with this condition and presents linearity in the concentration interval evaluated, which guarantees reliability for the ensuring of accurate results (Figure 6).

Finally, to assess whether the hemagglutination inhibition assay has the ability to maintain its precision and accuracy in the face of variations in the established operating conditions, a robustness test was conducted by modifying the interaction time between the serum samples and the NDV antigen. The results obtained demonstrate that the assay is robust and maintains its performance, as neither the CV% nor the % of recovery exceeded the acceptance criteria when the serum–antigen interaction times were changed (Table 4). Based on the results obtained in each of the tests, we conclude that the assay designed to evaluate the inhibitory capacity of human sera against the NDV antigen possesses the analytical attributes of consistency, reliability, and truthfulness in the results obtained, making it a useful tool for evaluating the immune response in serological studies.

## 4. Discussion

In this work, we report the different technical and analytical performance elements needed to develop and validate an HI serological assay that allows us to evaluate the level of antibodies against an NDV vaccine vector in human sera. Previous reports have described that it is possible to use RBCs obtained from the same species as the sera to be analyzed by HI or in combination with those of other species as part of a reading system for tests [6]. In line with this, to obtain the optimal conditions to establish the assay robustly and reproducibly, we tested combinations between RBCs from three different species and sera belonging to these same species, intending to implement and validate the assay in an easy, fast, and reliable way aligned with the international norms of adequacy and validation for analytical methods, under the criteria of ISO17025:2017. Based on the results obtained and considering factors such as the availability, traceability, and stability of the biological material, we used chicken RBCs, obtained from animals specifically destined for this purpose. In the standardized tests, we observed that chicken RBCs settle in an adequate time and present an easily observable shifting pattern compared to the tested mammalian RBCs, which ensures that we can assess their integrity and functionality, guaranteeing that we can define samples with subtle changes in inhibitory activity more precisely. During standardization, we also evaluated the use of pretreatments to eliminate potential nonspecific inhibitors of hemagglutination present in human serum samples. Sera pretreatments such as heating the sample at temperatures above 65 °C, pre-adsorption with RBCs, and mixing the sample with kaolin or RDE solutions have been reported [17]; therefore, we evaluated some of these treatments both individually and in combination, aiming to optimize and observe a complete agglutination pattern or eliminate possible interferences in the reading. However, we did not observe substantial differences between the use of kaolin and RDEs for the pretreatment of 5% chicken RBCs, as reported by another working group [16], with the advantage that the 5% chicken RBC sample shows minor dilution. Similarly, treatments with kaolin or RDEs dilute the sample ten-fold in the first dilution, making it impossible to detect low antibody titers, which is a disadvantage for the assay if it aims to identify the smallest potential response induced by the NDV vaccine vector. It is known, at least from one report, that the titers of people with the virus are not higher than 1:32 [11]. In another report by Boliar et al., 2006, it was indicated that these pretreatments do not have an effect on the correct determination of titers in the HI assay [16]. Meanwhile, the pre-adsorption treatment with chicken RBC suspension results in a two-fold dilution of the sample and does not interfere with the assay reading. We found several discrepancies concerning the dilution values for the reported samples; they do not agree with the experimental design described, suggesting a conceptual discordance between proportion and dilution. Therefore, in our opinion, it is not trivial to omit the working dilution [16,26]. To address the above, we considered the use of RBCs in carrying out the HI assay to adsorb the samples and reduce nonspecific agglutination; these conditions were used to perform method validation. According to the guidelines of the Eurachem guide, analytical methods adopted from standards or a commercial system require verification to ensure correct implementation in the laboratory [21]. However, when modifications are made to an existing method or the methodology is developed internally, a validation process is necessary. For this reason, we submitted our modified method to a validation process, aiming to ensure that the results provided were accurate, reliable, and truthful for the analysis of human sera [22]. Selectivity, sensitivity, specificity, PPV, NPV, PDR, and NDR are qualitative parameters that determine the degree of interference of the matrix in the assay and evaluate the probability of obtaining positive or negative results when anti-NDV antibodies are present or absent [21]. The parameters considered in the validation showed that the assay is specific and sensitive for the detection of positive or negative samples under the tested conditions. Accuracy was evaluated by analyzing repeatability and intermediate precision. Repeatability allows the evaluation of the variability in results obtained under practically identical conditions within short periods or with the same analyst, while intermediate precision allows the evaluation of variability between different days, analysts, or equipment, respectively [22]. The results indicate that the method is accurate, as no significant variation was observed in the conditions evaluated in our laboratory; thus, the method meets the proposed use objective. However, it is essential to recognize that the validation of the test is limited by the absence of reproducibility studies. While the publication of the International Conference on Harmonization of Technical Requirements for Registration of Pharmaceuticals for Human Use (ICH) established reproducibility as an essential parameter to evaluate tests conducted in two different laboratories, the results obtained suggest that the method is suitable for use in our specific environment [22]. The results acquired in terms of the accuracy and linearity of the method were consistent with those reported by Morokutti and collaborators [5] in a modified HI test to evaluate antibodies against the influenza virus. Finally, our results demonstrated that the interaction time between the NDV antigen and the serum sample, within a range of 15 to 45 min of incubation, does not significantly influence the test results. This provides flexibility for the method, making it an easy, fast, and effective tool for detecting antibodies against NDV in human serum from individuals vaccinated with vaccines based on NDV or those who received a prophylactic and therapeutic treatment based on NDV [27]. Previous studies with anti-vector antibodies in adenoviral platforms demonstrated the method’s utility for elucidating the effects of homologous or heterologous immunization regimens, concluding that homologous regimens decrease antibody production against a target antigen, while heterologous regimens favor a decrease in anti-vector antibodies promoting the production of the antigen of interest [28,29]. Therefore, the use of new vaccines, including the NDV viral vector and other vectors, has great potential and makes it necessary to have sensitive and validated assays that allow the identification of the production of neutralizing antibodies against NDV in clinical studies where this vector is used. Additionally, the experimental validation of the HI assay is essential to guarantee the reliability of results developed in research laboratories. This supports the request for sanitary registration before the competent authority, requested through a Common Technical Document (CTD) developed by the ICH [30].

## 5. Conclusions

The tests carried out for the validation of the serological assay “Method of Analysis to Evaluate the Inhibitory Capacity of Human Sera with the Hemagglutination Inhibition (HI) Technique against Newcastle Disease Antigen” demonstrated that the method is statistically selective, sensitive, and specific, indicating high diagnostic reliability, as well as satisfactory results in the acceptance criteria of positive or negative predictive value. The method is accurate and precise, and it is observed that there is a linear correspondence between the expected titer value and the observed titer value, with the method also being robust against the evaluated variations. These variations did not affect the results of evaluating the inhibitory capacity of human sera with the HI technique for the NDV antigen. Therefore, we conclude that the suitability of the analytical method for its intended use is confirmed and that the method guarantees the reliability of results obtained under the established operating conditions.

## Figures and Tables

**Table 1:** Summary of tested conditions for standardization of RBCs sedimentation and “teardrop” formation.

Temperature 17–19 °C				Temperature 20–25 °C			
RBC	[%]	Sedimentation Time	Teardrop Formation	RBC	[%]	Sedimentation Time	Teardrop Formation
**Chicken**	1	35 min	40 s	**Chicken**	1	30 min	30 s
	1.3	35 min	40 s		1.3	30 min	25 s
	2	30 min	30 s		2	30 min	20 s
**Human**	1	50 min	No	**Human**	1	50 min	No
	1.3	50 min	No		1.3	50 min	No
	2	50 min	No		2	50 min	No
**Pig**	1	>4 h	No	**Pig**	1	>3 h	No
	1.3	>4 h	No		1.3	>3 h	No
	2	>3 h	No		2	>3 h	No

**Table 2:** Statistical tests to evaluate the analytical performance of the HI assay: sensitivity, specificity, PPV, and NPV and acceptance criteria.

Present			Absent			
Positive *	(A) True positives	20.00	(B) False positives	0.00	A + B	20.00
Negative *	(C) False negatives	0.00	(D) True negatives	20.00	C + D	20.00
A + C		20.00	B + D	20.00	A + B + C + D	40.00
**Sensitivity = [A/(A + C)]**		1.00	Criteria:	≥0.95		
			Result:	1.00	≥	0.95
			Conclusion:	**Approved**		
**Specificity = [D/(D + B)]**		1.00	Criteria:	≥0.95		
			Result:	1.00	≥	0.95
			Conclusion:	**Approved**		
**Positive Predictive Value = [A/(A + B)]**		1.00	Criteria:	≥0.95		
			Result:	1.00	≥	0.95
			Conclusion:	**Approved**		
**Negative Predictive Value = [D/(C + D)]**		1.00	Criteria:	≥0.95		
			Result:	1.00	≥	0.95
			Conclusion:	**Approved**		

* The values were obtained from the experimental procedures.

**Table 3:** Statistical tests to evaluate the analytical performance of the HI assay: PDR and NDR and acceptance criteria.

Present			Absent			
Positive *	(A) True positives	20.00	(B) False positives	0.00	G = A + B	20.00
Negative *	(C) False negatives	0.00	(D) True negatives	20.00	H = C + D	20.00
E = A + C		20.00	F = B + D	20.00	A + B + C + D	40.00
**Positive diagnostic reliability = A/G**		1.00	Criteria:	≥0.95		
			Result:	1	≥	0.95
			Conclusion:	**Approved**		
**Negative diagnostic reliability = C/H**		0.00	Criteria:	<0.05		
			Result:	0	<	0.05
			Conclusion:	**Approved**		

* The values were obtained from the experimental procedures.

**Table 4:** Summary of results obtained from HI assay to determine robustness. Robustness is a parameter that evaluates the influence of noise variations such as the interaction time between serum samples and the NDV antigen; 30 replicates of a sample of human serum enriched with positive pig serum were used, with a calculated HI titer value of 1:128, and subjected to three different antigen–sample incubation times (15, 30 and 45 min). Each result was obtained from samples evaluated in triplicate.

Assay Robustness				
Conditions	Results			
Variarion: interaction time (serum/antigen) % Recovery (Observed titer/Expected titer) × 100	Time (min)	**15**	**30**	**45**
	Criteria:	80 to 120%		
	Result:	100	110	106
CV% (σ/Mean) × 100	Criteria:	≤20%		
	Result:	0%	6%	7%
	Conclusion:	**Approved**	**Approved**	**Approved**

## Data Availability

The data presented in this study are available upon request from the corresponding author.
